# Interstitial Telomeric-like Repeats (ITR) in Seed Plants as Assessed by Molecular Cytogenetic Techniques: A Review

**DOI:** 10.3390/plants10112541

**Published:** 2021-11-22

**Authors:** Alexis J. Maravilla, Marcela Rosato, Josep A. Rosselló

**Affiliations:** Jardín Botánico, ICBiBE, Universitat de València, c/Quart 80, E-46008 València, Spain; maravil3@alumni.uv.es (A.J.M.); marcela.rosato@uv.es (M.R.)

**Keywords:** interstitial telomeric repeats, in situ hybridisation, chromosomal landmarks, karyological evolution

## Abstract

The discovery of telomeric repeats in interstitial regions of plant chromosomes (ITRs) through molecular cytogenetic techniques was achieved several decades ago. However, the information is scattered and has not been critically evaluated from an evolutionary perspective. Based on the analysis of currently available data, it is shown that ITRs are widespread in major evolutionary lineages sampled. However, their presence has been detected in only 45.6% of the analysed families, 26.7% of the sampled genera, and in 23.8% of the studied species. The number of ITR sites greatly varies among congeneric species and higher taxonomic units, and range from one to 72 signals. ITR signals mostly occurs as homozygous loci in most species, however, odd numbers of ITR sites reflecting a hemizygous state have been reported in both gymnosperm and angiosperm groups. Overall, the presence of ITRs appears to be poor predictors of phylogenetic and taxonomic relatedness at most hierarchical levels. The presence of ITRs and the number of sites are not significantly associated to the number of chromosomes. The longitudinal distribution of ITR sites along the chromosome arms indicates that more than half of the ITR presences are between proximal and terminal locations (49.5%), followed by proximal (29.0%) and centromeric (21.5%) arm regions. Intraspecific variation concerning ITR site number, chromosomal locations, and the differential presence on homologous chromosome pairs has been reported in unrelated groups, even at the population level. This hypervariability and dynamism may have likely been overlooked in many lineages due to the very low sample sizes often used in cytogenetic studies.

## 1. Introduction

The physical package of genetic material is organised in universal structures called chromosomes. In prokaryotes, and in the organelles, chromosomes display a single and circular structure in the absence of a surrounding membrane envelope. However, in the nucleus of most eukaryotes, chromosomes are linear, and their numbers, shape, size, and C-genome size vary greatly among species. 

**Figure 1:**
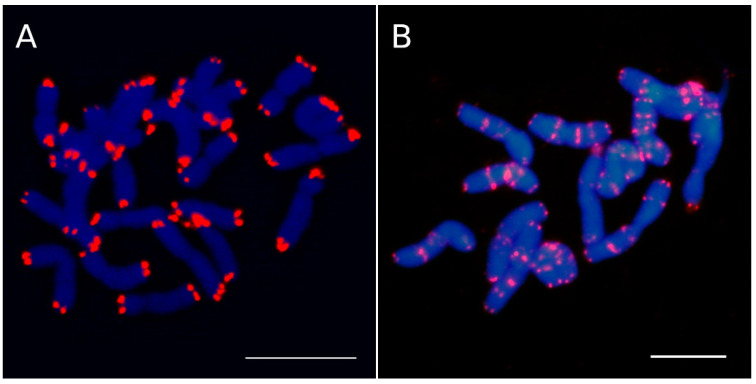
Telomeric sequences (*Arabidopsis*-type repeats) in (**A**) *Lysimachia minoricensis* (Primulaceae) and (**B**) *Anacyclus pyrethrum* (Asteraceae)*. L. minoricensis* lacks ITR repeats, whereas *A. pyrethrum* shows many ITR sites located at proximal and interstitial regions. Scale bars = 10 µm.

Structurally, a canonical eukaryote chromosome consists basically of chromatids, a centromere, and telomeres, which are preserved during cell division through mitosis and meiosis. Centromeres and telomeres are vital for the integrity of eukaryotic chromosomes. The former play a key role in the precise segregation of chromosomes throughout mitosis and meiosis processes during cell divisions. Meanwhile, telomeres are the terminal DNA-nucleoprotein complexes of chromosomes (Figure 1A), capping their ends and protecting the chromosome against end-to-end fusions [1]. 

Interstitial telomeric repeat (ITR) sites, also known as interstitial telomeric sequences (ITSs), consist of tandem repeats of telomeric motifs that are located within intrachromosomal regions (Figure 1B), including repeats located close to the centromeres and the ones found between the centromeres and the telomeres [2]. 

Their presence in fungi [3], vertebrates [4,5], and plants [6,7,8,9,10] suggests that (1) the acquisition of telomeric repeats inside chromosomes in unrelated organisms is a convergent event during karyotype evolution, and (2) multiple cytogenetic and molecular mechanisms might have contributed to the diversity of their formation. 

Discovering telomeric repeats in interstitial regions of plant chromosomes using molecular cytogenetic techniques was achieved several decades ago [11], and many subsequent reports have been published to date e.g., [12]. Their study has revealed the perception that they are relevant and stable karyological landmarks. However, the information is scattered and has not been critically evaluated from an evolutionary perspective. To fill these gaps, we have critically reviewed the available information related to ITRs on seed plants as assessed by molecular cytogenetic methods. Based on the currently available data, these karyological landmarks are evaluated by (1) detecting emerging patterns of variation in presence and site number across major evolutionary lineages and at several hierarchical levels, (2) assessing the possible associations between the occurrence, number and chromosomal location of ITR sites and the chromosome number of the analysed species, (3) estimating the utility of ITRs as phylogenetic and taxonomic markers in plants, (4) discussing the main mechanisms involved in the genesis of ITRs, and (5) assessing the role of biological processes that may have triggered the evolution of ITRs in seed plants. 

## 2. Molecular Cytogenetic Approaches Used in ITR Detection

In situ hybridisation (ISH) techniques have become one of the most powerful approaches for mapping specific sequences of DNA in plant cytogenetics, including telomere sequences. The basic principles underlying ISH is similar among all the types of experimental variants that have been developed, regardless of whether standard or sophisticated methods were used. However, experimental issues involving the type of the used probes (cloned, synthetic oligonucleotides), probe labeling (nick-translation, PCR-labeling, pre-labeled oligomer), and probe detection (fluorescent, enzymatic) contribute to the sensitivity of ISH approaches [13,14,15,16]. Several technical approaches have been used to date to detect telomeric repeats in plants, including non-Isotopic ISH [11], FISH (fluorescent in situ hybridisation; [6]), PRINS (primer in situ DNA labeling, [17]), PNA-FISH (peptide nucleic acid-FISH, [18]), ND-FISH (non-denaturant-FISH, [19]), PLOPs-FISH (Pre-Labelled Oligomer Probes, [16]), and CO-FISH (chromosome orientation-FISH, [20]). The drawback of molecular cytogenetic methods is that short arrays of telomeric-like sites may be undetectable by ISH [21]. In these cases, DNA sequencing of interstitial chromosomal regions or whole genomes is the best available option [22]. 

Initially, the location of telomeres in plant chromosomes were identified in *Hordeum vulgare* and *Secale cereale* by [11], who also detected interstitial sites. Some years later, a synthetic oligonucleotide (TTTAGGG), representing the canonical *Arabidopsis*-type repeat was used as a template in PCR and fluorescently labelled to locate telomere repeats in several unrelated flowering plant species [6]. Most of the studies (71.56%) dealing with the cytogenetic mapping of plant telomeres used synthetic oligonucleotide probes for ISH, including the *Arabidopsis*-type repeat (53.13%; e.g., [7]), the vertebrate-type repeat (TTAGGG) (15.62%; e.g., [23]), other unusual plant-specific telomere sequences (CTCGGTTATGG, TTTTTTAGGG, T_4_-_5_AGCA, TTCAGG and TTTCAGG; 2.14%, e.g., [24,25,26,27]), and the *Tetrahymena*-type repeat (TTGGGG; 0.67%, e.g., [23]), while a significantly lower number (28.44%) used cloned sequences involving the *Arabidopsis* clone (27.90%; e.g., [28]) or other specific telomeric regions (0.54%; e.g., [18]). The most likely reasons explaining the preferential use of synthetic probes over cloned sequences may be related to the more complex technical requirements and higher costs involved in the handling and conservation of clones. Currently, ITR repeats detected in plants are constituted by the *Arabidopsis*-type (TTTAGGG), vertebrate-type (TTAGGG), and *Cestrum*-type (T_4_-_5_AGCA) sequences. 

## 3. ITR Sampling in Seed Plants

A total 627 species from 330 genera belonging to 79 families (*sensu* APG IV) have been karyologically analysed to detect telomeric sequences in seed plants [6,7,8,10,11,12,16,17,18,19,20,23,24,25,26,27,28,29,30,31,32,33,34,35,36,37,38,39,40,41,42,43,44,45,46,47,48,49,50,51,52,53,54,55,56,57,58,59,60,61,62,63,64,65,66,67,68,69,70,71,72,73,74,75,76,77,78,79,80,81,82,83,84,85,86,87,88,89,90,91,92,93,94,95,96,97,98,99,100,101,102,103,104,105,106,107,108,109,110,111,112,113,114,115,116,117,118,119,120,121,122,123,124,125,126,127,128,129,130,131,132,133,134,135,136,137,138,139,140,141,142,143,144,145,146,147,148,149,150,151,152,153,154,155,156,157,158,159,160,161,162,163,164,165,166,167,168,169,170,171,172,173,174,175,176,177,178,179,180,181,182,183,184,185]. These figures sharply contrast with the greater amount of data reported for nuclear ribosomal DNA loci (35S and 5S rDNA families), the most popular chromosomal landmarks used in plant molecular cytogenetics, with data available for 2148 species, 540 genera, and 114 families [186]. 

**Figure 2:**
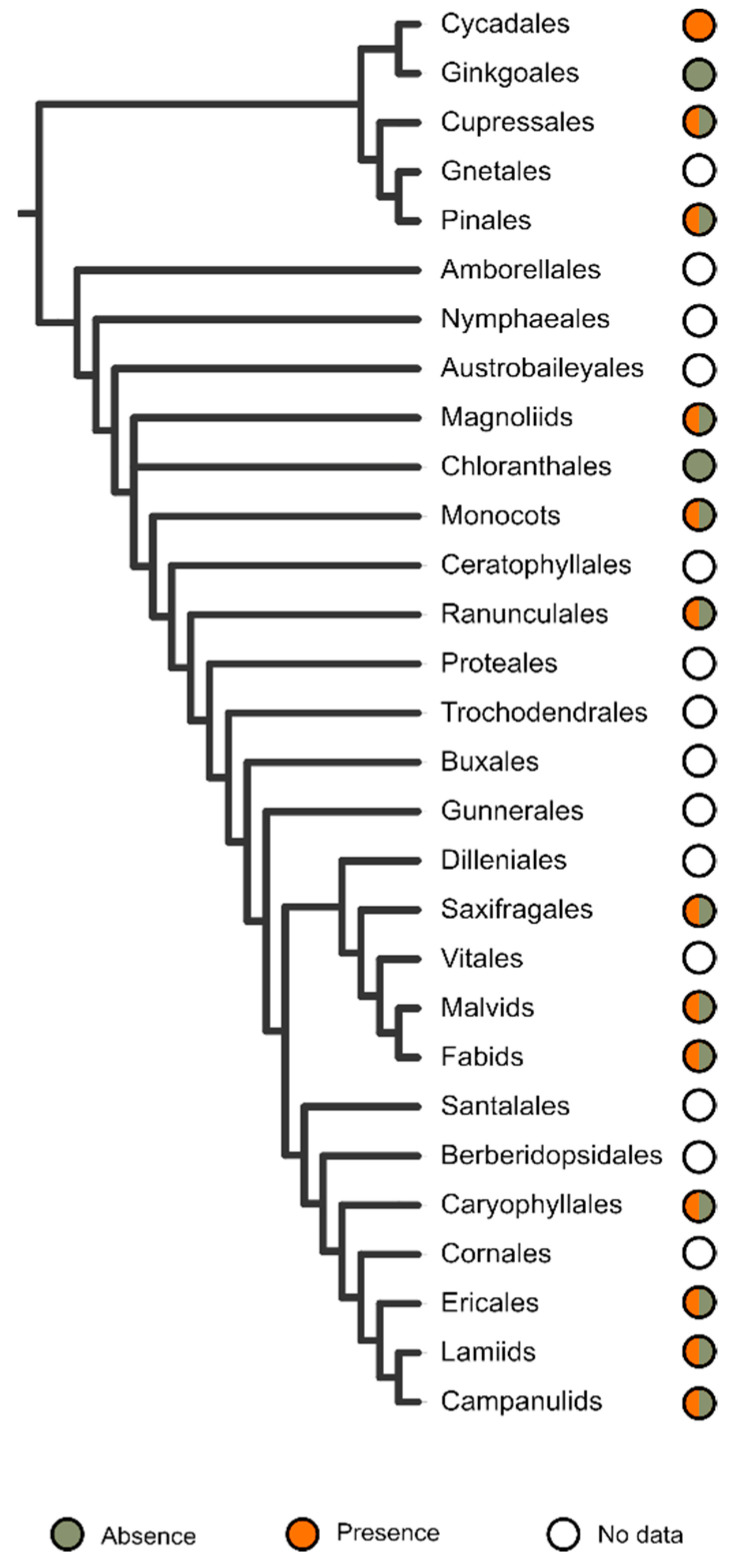
Occurrence of ITR sites in seed plants. The absence (grey colour) and presence (orange colour) in major lineages is illustrated. Unsampled groups are depicted in white. The overview of the phylogenetic relationships is based on [188] for Gymnosperms and [189] for Angiosperms.

The sampling for detecting telomeric sequences is uneven and biased towards the analysis of large groups, with some exceptions (Figure 2). 

Whereas in gymnosperms there is a lack of data only for Gnetales, in angiosperms the number of major groups analysed (11) nearly equals those for which there is no data (13). Speciose ordinal groups not sampled to date are few and include Proteales, Vitales, Santalales, and Cornales, which encompass between 14–151 genera and 590–1750 species [187]. Unfortunately, no species from the three most basal lineages of angiosperms (Amboreallales, Nymphaeales, Austrobaileyales) have been analysed. Although the overall diversity of these orders is fairly limited (12 genera and about 175 species; [187]), their key position in the ancestral diversification of flowering plants makes them priority targets for assessing the presence of ITRs. 

## 4. Taxonomic Distribution of ITRs Is Widespread among Major Lineages of Seed Plants

**Figure 3:**
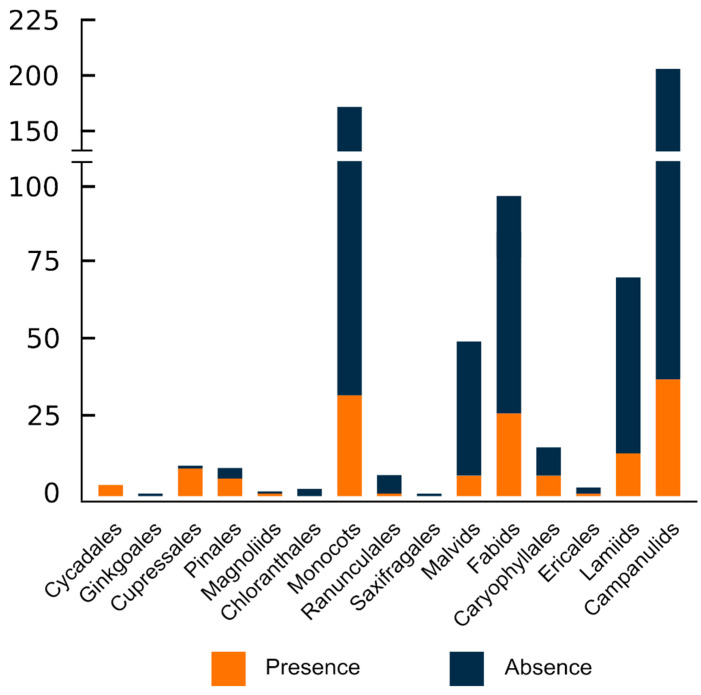
Taxonomic distribution of ITRs in the sampled lineages of seed plants. The number of recorded species is indicated for each group (orange colour). The circumscription of higher taxonomic lineages follows the hypothesis of the Angiosperm Phylogeny Website [187].

With the exception of Chloranthales, which exhibits a scarce diversity and has only had three of its analysed, and the monotypic Ginkgoales, all major evolutionary groups sampled had ITRs in their karyotypes (Figure 2 and Figure 3). 

However, heterogeneity regarding the distribution at lower taxonomic units is noteworthy. Thus, ITRs occur in only 36 out of 79 sampled families (45.57%), suggesting that disparate results occur within major plant lineages. In gymnosperms, the families with the higher number of ITR occurrences are Podocarpaceae (8 spp.) and Pinaceae (6 spp.), whereas Asteraceae (37 spp.), Fabaceae (21 spp.), and Poaceae (8 spp.) lead among angiosperms. It should be stressed, however, that due to the uneven sampling effort made at different taxonomic levels, these results could be skewed and may not reflect the real values. In this regard, it is worth mentioning that at the family level, the number of species showing ITRs in their karyotypes is strongly correlated to the number of sampled species (Pearson correlation value = 0.994, *p* < 0.0001).

Overall, lower and similar frequencies of occurrence are attained at the generic and species level. ITRs have been detected in only 88 out of 330 analysed genera (26.67%) and in 149 out of 627 sampled species (23.73%). These results clearly show that although ITRs are widespread in seed plants, their frequency at low taxonomic units is fairly moderate. 

## 5. ITRs Preferentially Occur at Interstitial Chromosomal Arms 

**Figure 4:**
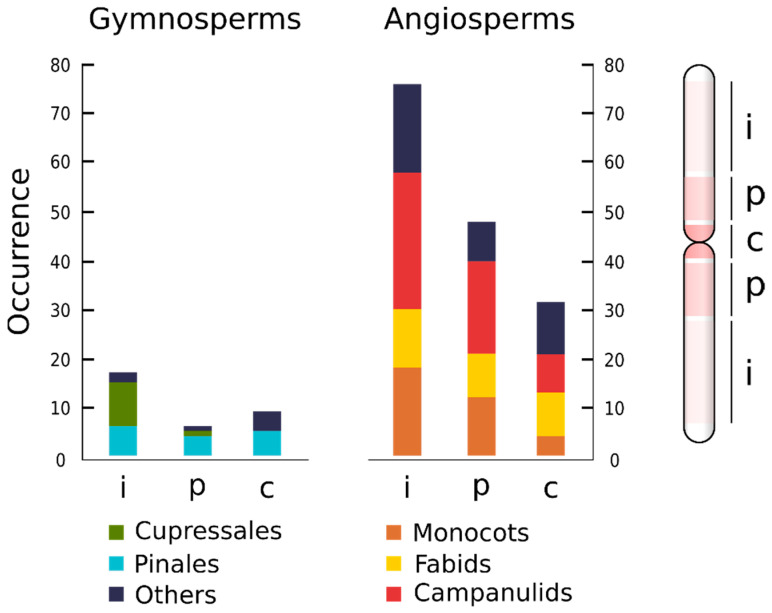
Longitudinal distribution of ITR sites along the chromosome arm in major lineages of gymnosperms and angiosperms. The percentage of occurrence in each chromosomal region is indicated. The chromosome arm was divided into three major domains of unequal size, i.e., centromeric (c), proximal (p), and interstitial (i).

The longitudinal distribution of ITR sites along the chromosome arms is uneven (Table 1). Overall, more than half of the ITRs occur between proximal and terminal locations (49.5%), followed by proximal (29.0%) and centromeric (21.5%) arm regions. Interestingly, the relative order of occurrence contrasts between gymnosperms and angiosperms. The former shows higher occurrences at centromeric locations whereas proximal locations are more abundant in angiosperms (Figure 4). 

Within gymnosperms there are several contrasting trends for Pinales and Cupressales orders (Figure 4). No centromeric ITR signals have been reported for the latter and most occurrences are at interstitial sites. Dissimilar values are reported for Pinales, where nearly equivalent occurrences at interstitial, proximal, and centromeric regions are reported. In angiosperms, the three major lineages with the highest number of species with ITRs (Monocots, Fabids and Campanulids) do not show clear divergent patterns related to the longitudinal distribution of ITR sites along the chromosome arms (Figure 3). 

## 6. The Number of ITR Sites Greatly Varies among Congeneric Species and Higher Taxonomic Units

There is a wide range of reported ITR signals, from one (*Gibasis pulchella*, Comme-linaceae, [6]) to 72 signals (*Pinus taeda*, Pinaceae; [35]), although a high but undetermined number reported in the monocot *Anthurium wendlingeri* [84] may eventually exceed the later value. ITR signals mostly occur as homozygous loci in most species. However, odd numbers of ITR sites reflecting a hemizygous state have been reported in both gymnosperm and angiosperm groups. Hemizygosity in the former is restricted to Cycadales (*Cycas revoluta* [30]. Interestingly, *Cycas revoluta* is a dioicous species, and Hizume et al. [30] indicated that hemizygosity was associated with the heteromorphic sexual chromosome pair. Thus, male plants showed an odd number of ITR signals when compared to females, providing additional cytogenetic markers to characterize sexual chromosomes in this species. The differential association of ITR sites and sexual chromosomes has not been reported in other species of Cycadales which have male and female individuals [37,38]. Unfortunately, neither the number of individuals analysed nor the gender of their accessions were specified [37,38]. This casts doubt on whether ITRs are involved in the genomic evolution of the sexual chromosomes in Cycadales. 

Greater hemizygosity is present in angiosperms (16 species), including Campanulids (nine species), Monocots (four species), Fabids (two species), and Caryophyllales (one species). All sampled species are hermaphrodite or monoicous and the odd numbers of ITR are not related to any obvious biological trait.

**Figure 5:**
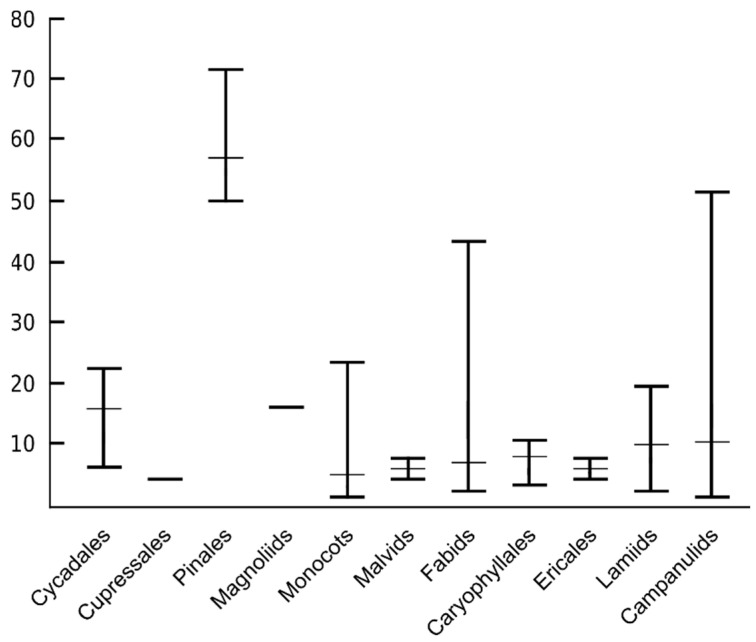
Number of ITR sites reported for the major lineages of seed plants. The range and average number of ITR signals are represented.

The overall average number of sites in species where ITRs have been reported is 10.33 ± 13.36. However, when the values are separately calculated for gymnosperms and angiosperms, the data strongly differ. Gymnosperm species show an average of 32.60 ± 24.53 sites in contrast with the exceedingly low value of 7.82 ± 8.50 obtained from angiosperms. Moreover, there are also diverging values in ITR site numbers among major lineages of seed plants. This has been clearly revealed in groups where a substantial number of species showing ITRs have been reported, e.g., Monocots, Fabids, Lamids, and Campanulids (Figure 5). 

Congeneric species may differ in the presence or absence of ITR signals as observed in unrelated groups from 16 families. This is illustrated in Asteraceae, where intrageneric polymorphisms have been detected in *Achillea*, *Anacyclus*, *Anthemis*, *Cladanthus*, *Nassauvia*, and *Sonchus* [69,82], Alstroemeriaceae (*Alstroemeria*, [31,44]), Brassicaceae (*Brassica*, [60]), Solanaceae (*Cestrum*, [26,87]), Rutaceae (*Citrus*, [92,93]), Poaceae (*Colpodium*, *Hordeum*, [47,61,94,95]), Cucurbitaceae (*Cucumis*, [98,99]), Orchidaceae (*Dendrobium*, [41]), Cyperaceae (*Eleocharis*, [48,97]), Lentibulariaceae (*Genlisea*, [27]), Cannabaceae (*Humulus*, [100,101]), Juncaceae (*Luzula*, [96]), Amaryllidaceae (*Nothoscordum*, *Prospero*, [18,23,86]), Rosaceae (*Rosa*, [62,103]), Fabaceae (*Senna*, *Vicia*, [7,45,74]), and Commelinaceae (*Tradescantia*, [6,103]). 

## 7. Variable Presence and Location of ITR Sites Occur within Species 

Intraspecific variation regarding the presence and absence of ITR signals has been reported in a relatively low number of specie: the gymnosperm *Zamia furfuracea* (Zamiaceae, [7,37]) and the angiosperms *Beta vulgaris* (Amaranthaceae, [7,104]), *Brassica oleracea* (Brassicaceae [60,102]), *Cestrum parqui* (Solanaceae, [26,87]), *Hordeum vulgare* (Poaceae, [6,11]), *Humulus scandens* (Cannabaceae, [56,100]), *Luzula luzuloides* (Poaceae, [7,96]) *Vicia faba* (Fabaceae, [7,45]), *Solanum tuberosum* (Solanaceae, [7,31]), *Sonchus tenerrimus* (Asteraceae, [69]), *Tanacetum parthenium* (Asteraceae, [69,103]) and three species of *Anacyclus* (Asteraceae)*, A. clavatus, A. monanthos* and *A. valentinus* [82]. 

Moreover, additional intraspecific polymorphisms involving contrasting chromosomal locations and a differential presence of the ITR sites on homologous chromosome pairs have been also reported. The most extreme case documented thus far occurs in *Anacyclus* (Asteraceae), where intraspecific hypervariability and dynamism was documented in six of the nine known species of the genus, using a large sampling size [82]. The extent of the variation was so high that all analysed individuals showing ITRs could be distinguished by their cytogenetic patterns [82]. Such remarkably high levels of polymorphism, indicating that ITRs are a labile genomic feature within a single species, were unnoticed and not previously reported in plants. An issue in need of additional research is to assess whether *Anacyclus* is an isolated case of ITR dynamism in seed plants or if this pattern might have been overlooked in other unrelated groups due to the low sample sizes often used in cytogenetic studies.

## 8. The Presence of ITRs and the Number of Sites Is not Significantly Related to Number of Chromosomes 

Overall, no apparent association was detected between the presence of ITR signals and the number of chromosome of the analysed species (Pearson correlation value r = −0.130, *p* < 0.001). This lack of association was also reported when analysing partial but significant datasets. Thus, for Asteraceae (by far the most sampled family of seed plants), the correlation value between these two parameters is close to 0 [69]. 

**Figure 6:**
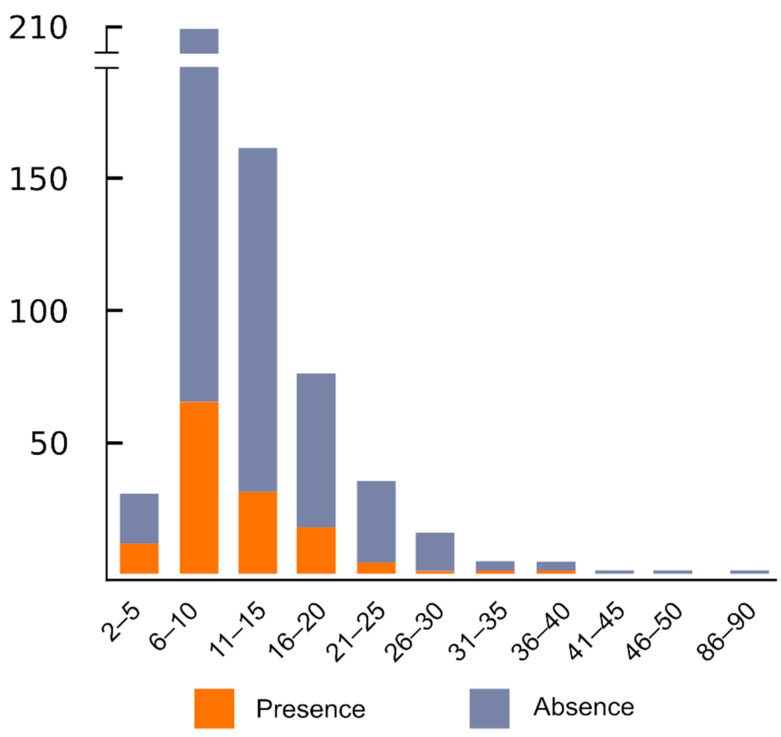
Presence and absence of ITR sites in seed plants according to the haploid chromosome number (*n*) of the analysed species.

ITRs were detected in all haploid chromosomal intervals from *n* = 2–5 up to *n* = 36–40, albeit with contrasting values (Figure 6). 

The existence of ITRs has not been documented in species showing a haploid chromosome number higher than *n* = 41. The maximum presence of species showing ITRs falls within chromosome interval *n* = 6–10. The latter results should be interpreted with caution and probably have no underlying evolutionary significance. A close dissection of the data indicates that 39.4% of the species from the *n* = 6–10 range showing ITRs belong to the Asteraceae where the highest presence of ITRs occurs in species with *n* = 9 chromosomes [69]. 

As a rule, few species with a chromosome number *n* = 21 or higher have been reported to show ITRs. However, it should be noted that this trend may be another result of the sampling since very few species with high chromosome numbers have been analysed. 

Similarly, no significant association is detected between the total ITR sites present in the chromosome complement and the chromosome number (Pearson correlation value r = 0.031, *p* = 0.6745). This is clearly evidenced in Asteraceae, where species sharing the same number of chromosomes (2*n* = 18) showed a wide range of ITR sites (range = 2–52; [69]). Almost all fundamental lineages of seed plants have experienced episodes of whole-genome duplication events [190]. The recurrent cycles of paleo-polyploidisation have been followed by massive genomic and chromosome rearrangements resulting in losses, amplifications, translocations and inversions of DNA fragments thereby modifying genome architecture and ancestral chromosome numbers [191,192]. These complex and ancient evolutionary scenarios might compromise the right interpretation of karyotype changes and hypotheses on chromosomal number evolution, explaining the lack of positive correlation between ITRs and chromosome number.

## 9. Origin of ITRs

Since the initial identification in plant karyotypes it was suggested that ITRs could be considered cytological landmarks of chromosomal rearrangements [7]. It has been hypothesised that the presence of ITRs in the centromeric and peri-centromeric regions illustrate could be vestiges of ancestral end-to-end fusion events between non-homologous chromosomes that caused descendent dysploidy [84,85,193,194,195]. However, the presence of ITRs could also be explained by chromosome translocation and inversion mechanisms [196]. Thus, translocation-based descending dysploidy associated to the occurrence of ITRs has been suggested in Brassicaceae [197], and the presence of ITRs in *Phaseolus microcarpus* may be generated through pericentric inversions [185]. Another possible causal mechanism based on the heterochomatin distribution model [198] is the equilocal dispersion of telomeric DNA to interstitial regions of the chromosome via transposition or heterologous recombination [86].

Telomeric sequences are in fact a type of microsatellite repeats regarding their length, high number of copies, and their disposition in tandem arrays in the genome. This way, the amplification of telomere repeats could be generated through similar mechanisms involved in the genomic evolutionary dynamics of the satellite DNA [199]. The rapid turnover of satellite DNA and the amplification of long stretches of repetitive sequences could be explained by a mechanism of rolling-circle replication of extrachromosomal circular DNA (eccDNA) [200,201]. In fact, it has been suggested that eccDNA could be involved in the occurrence and amplification of megabase-sized ITRs in *Solanum* [58] and the hypervariable ITR distribution in *Anacyclus* species [82]. Alternatively, the repair mechanisms of DNA double-strand breaks could also help explain the presence of short stretches of ITRs [2]. The above mechanisms are not mutually exclusive and might act jointly to achieve the evolutionary turnover of plant ITRs. The diversity of processes likely involved in the origin of ITRs illustrates the need for caution when assessing their role in karyological evolution, genome divergence, and the evolutionary history of plants. 

## 10. Data Analysis 

Relevant information was retrieved regarding the number of chromosomes, karyotype description, presence or absence of ITRs, their location on chromosome arms, the type of telomeric repeat and methodological approach, if available. When possible, it was produced from the raw data obtained from published research. Species showing ITRs were further analysed and the number of sites and their location along the chromosome were recorded. The chromosome arm was divided into three major domains of unequal size, i.e., centromeric (c), proximal (p), and interstitial (i); and the location of ITR sites was mapped on these chromosomal regions. Basically, this distinction is based on [202], except that no distinction was made between the interstitial-proximal and interstitial-terminal regions. In the case of holokinetic chromosomes, ITR signals were arbitrarily classified as interstitials. The detailed distribution of ITRs in each of the two chromosome arms was usually not provided or listed in the original publications. Accordingly, the locations of ITRs in each chromosome were pooled regardless of being present in the short or long arm. Species lacking detailed information regarding the precise number of ITR sites or their chromosomal distribution were not taken into account in the numerical analyses concerning these parameters. 

The current scientific name and authorships of the species covered in this paper were retrieved from [203]. The circumscription of the families and higher taxonomic lineages (orders) of seed plants follows the hypothesis of the Angiosperm Phylogeny Website [187]. The overview of the phylogenetic relationships among seed plants is based on [188] for Gymnosperms and [189] for Angiosperms. The information was connected in a single phylogenetic tree to map the presence of ITR data in an evolutionary context (Figure 1).

## 11. Conclusions

The detection of ITR sites using molecular cytogenetic techniques has provided relevant, but still limited, knowledge on the patterns and processes of plant evolution. The uneven taxonomic sampling performed to date is a major concern for obtaining a stable overall perspective on ITR evolution. Many lineages of angiosperms have not been analysed to assess the presence of ITRs. This is unfortunate, since there is a lack of data for critical groups, especially flowering plants, which are of paramount importance for assessing solid inferences about the ancestral states of ITRs in seed plants. 

Establishing the evolutionary trends in ITR evolution is further complicated by the emerging view that these telomeric sequences may show a remarkable intraspecific dynamism, even at the population level, involving site number, chromosomal locations and the differential presence of the ITR site on homologous chromosome pairs. In the absence of suitable and hierarchical sampling sizes (individuals, populations and species), this hypervariability and dynamism may have likely been overlooked in many groups. Unfortunately, most data available on plants are based on the unsatisfactorily low sample sizes commonly used in cytogenetic studies. The contention that ITRs are, contrary to previous hypotheses, a labile genomic feature within a species, may severely restrict their use as phylogenetic and taxonomic markers in plants. On the contrary, it may open new ways for applying ITRs as useful karyological markers at the population level, providing enough information to identify plant individuals and trace micro-evolutionary events. 

## Figures and Tables

**Table 1:** Longitudinal distribution of ITRs in seed plants. The percentage of occurrence in each chromosomal region is indicated. The chromosome arm was divided into three major domains of unequal size, i.e., centromeric (c), proximal (p), and interstitial (t).

	Seed Plants (%)	Gymnosperms (%)	Angiosperms (%)
c	21.5	28.1	20.1
p	29.0	18.8	31.2
i	49.5	53.1	48.7

## Data Availability

Not applicable.
